# Supplementary material for: Specific Cellular and Humoral Immune Responses to the Neoantigen RBD of SARS-CoV-2 in Patients with Primary and Secondary Immunodeficiency and Healthy Donors
Source: Biomedicines. 2023 Mar 28;11(4):1042. doi: 10.3390/biomedicines11041042 (PMC10135489; doi:10.3390/biomedicines11041042)
Supplement: Supplementary file 1 [file biomedicines-11-01042-s001.zip › biomedicines-2254317-supplementary.pdf]

Supplementary Table S1. Epidemiological and main clinical features of PID patients. RRTI: recurrent respiratory tract infections;

PID patient No.	Sex	Age	Main clinical features	IgRT
PID 1	F	41	RRTI, splenomegaly, bronchiectasis, insulin resistance	IVIG
PID 2	M	18	RRTI	SCIG
PID 3	M	55	RRTI, LGL leukemia	IVIG
PID 4	F	33	RRTI, bronchiectasis, splenomegaly,	IVIG
PID 5	F	30	RRTI	IVIG
PID 6	F	45	RRTI, chronic gastritis due to *H. pylori* eradicated in 2013, ferropenic anemia and T $CD8^{+}$ lymphopenia	IVIG
PID 7	M	42	RRTI, pancytopenia related to CMV infection and inflammatory disease	SCIG
PID 8	M	28	RRTI	SCIG
PID 9	F	69	RRTI, TTP	SCIG
PID 10	M	57	RRTI, hypothyroidism	IVIG
PID 11	F	65	RRTI, lymphoid granulomatosis with interstitial lung involvement, received treatment with rituximab (2016 to 2017) with secondary leukopenia, pigmented purpuric dermatosis	IVIG
PID 12	F	74	RRTI	IVIG
PID 13	F	71	RRTI	IVIG
PID 14	F	67	RRTI, chronic gastritis due to *H. pylori*	IVIG
PID 15	M	47	RRTI, chronic gastritis with intestinal metaplasia due to H. pylori eradicated in 2015	IVIG
PID 16	F	30	RRTI	No
PID 17	F	58	RRTI, TTP, splenomegaly, APS	IVIG
PID 18	F	62	RRTI, inverted psoriasis, polyarthralgia, $CD4^{+}$ T lymphopenia	SCIG
PID 19	F	45	RRTI, UTI, $CD4^{+}$ T lymphopenia	SCIG
PID 20	F	59	RRTI, chronic gastritis associated with non-eradicated *H. pylory*, malabsorption syndrome, nodular lymphoid hyperplasia and hiatal hernia, recurrence of thyroid papillary cancer	IVIG

Supplementary Table S2. Epidemiological and main clinical features of SID patients. FCR: fludarabine, cyclophosphamide and rituximab; R-CHOP: rituximab, cyclophosphamide, hydroxydaunorubicin hydrochloride (doxorubicin hydrochloride), vincristine (Oncovin) and prednisone; BR: bendamustine and rituximab. ITP: immune thrombocytopenia; RRTI: recurrent respiratory tract infections; RUTI: recurrent urinary tract infections.

SID patient	Sex	Age (yrs)	Clinical history	Disease status	IgRT
SID 1	F	58	NHL, RRTI	Not treated	No
SID 2	F	85	NHL treated with FCR, RRTI, malabsorption syndrome	In complete remission	IVIG
SID 3	F	68	CLL, RRTI, hypothyroidism	Not treated	No
SID 4	M	73	MM, RRTI	Not treated	No
SID 5	M	72	CLL, RRTI	Not treated	IVIG
SID 6	F	61	CLL, RRTI, asthma, RUTI	Not treated	IVIG
SID 7	F	66	MGUS, RRTI, RUTI, ITP	Not treated	No
SID 8	F	68	NHL treated with *FCR, RRTI*	In complete remission	IVIG
SID 9	F	72	NHL treated with *FCR, RRTI*	In complete remission	IVIG
SID 10	F	67	Diffuse large B-cell lymphoma treated *with R-CHOP*, RRTI	In complete remission	IVIG
SID 11	F	66	Diffuse large B-cell lymphoma treated *with R-CHOP*, RRTI, lymphoid interstitial pneumonia	In complete remission	IVIG
SID 12	M	55	NHL treated with BR, RRTI	In complete remission	IVIG
SID 13	M	83	NHL treated with rituximab, RRTI, ITP, bronchiectasis	In complete remission	IVIG

SID 14	F	68	NHL treated with FCR, RRTI	In complete remission	IVIG
SID 15	M	49	Diffuse large B-cell lymphoma treated *with R-CHOP*, RRTI	In complete remission	IVIG
SID 16	F	79	CLL, RRTI	Not treated	IVIG
SID 17	F	67	Diffuse large B-cell lymphoma treated *with R-CHOP*, RRTI, hypothyrioidism	In complete remission	IVIG
SID 18	F	70	MGUS, RRTI, acute cholangitis by *Staphylococcus aureus*	Not treated	IVIG
SID 19	F	71	CLL, RRTI	Not treated	No
SID 20	F	60	NHL treated with rituximab, RRTI	In complete remission	IVIG

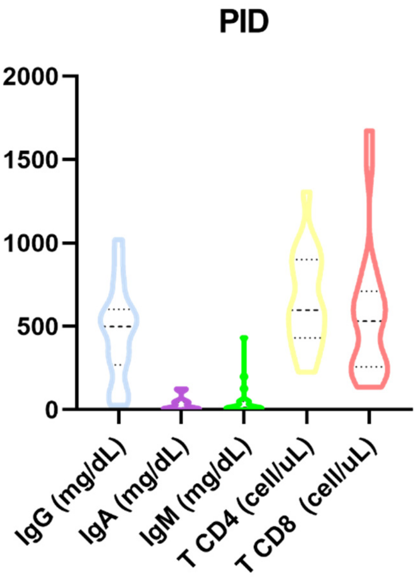

Supplementary Figure S1. Immunological features of the PID group.

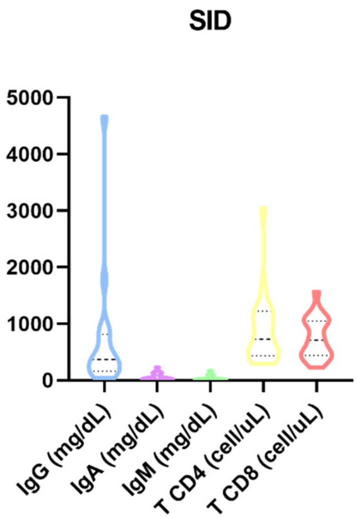

Supplementary Figure S2. Immunological features of the SID group.
